# Cost-effectiveness of protein-rich meals and snacks for increasing protein intake in older adults

**DOI:** 10.1016/j.jnha.2024.100381

**Published:** 2024-09-27

**Authors:** P. Rautakallio-Järvinen, S. Kunvik, M. Laaksonen, L. Fogelholm, I. Nykänen, U. Schwab

**Affiliations:** a School of Medicine, Institute of Public Health and Clinical Nutrition, University of Eastern Finland, Kuopio, Finland; b Satakunta Wellbeing County, Finland; c Fazer Lab, Oy Karl Fazer Ab, Finland; d Compass Group FS Finland Oy, Finland; e Department of Medicine, Endocrinology and Clinical Nutrition, Kuopio University Hospital, Wellbeing Services County of North Savo, Kuopio, Finland

**Keywords:** Older adults, Protein intake, Cost-effectiveness, Diet

## Abstract

**Objectives:**

To investigate the cost-effectiveness of protein-rich meals and snacks for increasing protein intake in home-dwelling older adults.

**Design:**

Cost effectiveness analysis from a randomized controlled trial, the Power Meals study.

**Setting:**

Participants were randomized into one of three groups for eight weeks: a protein-rich meal, snack and bread (Protein), a regular meal (Normal) and a control group without meal service (Control).

**Participants:**

Home-dwelling home care clients, caregivers and care recipients aged ≥65 years (n = 65).

**Measurements:**

Protein intake was assessed by a three-day food diary at the end of the study. Cost for the daily diet was estimated by using Finnish grocery store databases and the prices of the food service. The cost-effectiveness was assessed by an incremental cost-effectiveness ratio (ICER).

**Results:**

Costs for the daily diet in the Protein (8.35 €/d) and the Normal (7.94 €/d) groups were significantly higher than in the control group (5.65 €/d) (p < 0.001). Incremental cost-effectiveness analysis showed that increasing protein intake was cost-effective in the Protein group as incremental cost-effectiveness ratio was 8.11 in the Protein, 8.72 in the Normal and 6.45 in the Control group.

**Conclusions:**

Including protein rich meals and snacks in a diet increases protein intake in home-dwelling older adults cost-effectively.

## 1 Introduction

Malnutrition in older adults is mainly due to protein energy wasting [1]. Protein is needed for maintaining bone mass, muscle mass and muscle strength, and it can also protect against unintentional weight loss [2,3]. Protein intake is insufficient according to Finnish studies among home-dwelling older adults [[4], [5], [6]].

Dietary interventions based on a food-based protein fortification of a standard diet could be considered useful in older adults at risk of malnutrition [7]. Food-based nutrition interventions have been found to be effective at improving clinical outcomes associated with malnutrition [8]. They also have a low cost of implementation and may be cost-effective [8]. Increasing protein intake by personalized dietary advice has been found to be cost-effective [9]. Optimized meal services have also had economic benefits in terms of saved health care costs and improved quality of life [10]. Food costs can influence the diet quality as diets of higher nutrient density and nutritional quality tend to have higher costs [11]. Meal services have been reported to be effective in improving protein intake [12].

Our earlier analysis from the Power Meals study suggested that providing protein-rich foods and snacks can increase protein intake when comparing to the regular food service meals or having no meal service or dietary advice [13]. Therefore, we aimed to investigate the cost-effectiveness of increasing protein intake by protein-rich foods and snacks in home-dwelling older adults.

## 2 Materials and methods

### 2.1 Participants

This study is part of the Power Meals study from 2018 [13]. Power Meals was a randomized, controlled intervention study, which explored the effects of an 8-week home meal service on nutrient intake, physical performance and health-related quality of life in home-dwelling older adults. Power Meals had two primary outcomes, which were protein intake in g/ikg/d assessed by three-day food diary and physical performance as assessed by chair stand test in Short Physical Performance Battery (SPPB). Participants were recruited from home care clients or family caregivers and recipients in the Pori area in Finland, during a home care nurse`s visit or by phone. If they were interested, a nutritionist made a home visit during which information about the study was provided and informed consent obtained. The recruitment has been reported in detail earlier [13]. The study was approved by the Ethics Committee of the Hospital District of Southwest Finland.

There were 78 participants in the Power Meals study, of which 67 completed the study. Two of the participants did not fulfill the food diary at the end of the study, so in this cost-effectiveness analysis we had 65 participants. Most of the participants were home care clients (38.5 %), 32.3 % were caregivers, 27.7 % care recipients and 1.5 % were spouses of home care clients. Mean age of the participants was 78 ± 7 years.

### 2.2 Intervention and meals

Participants were randomized into three different groups (Protein, Normal or Control) as previously described [13]. The Protein group (PG) received one home-delivered meal, one protein-rich snack and two slices of protein-rich bread every day. The Normal group (NG) received one regular home-delivered meal every day. For PG, protein-rich meals were chosen from the food service’s meal selection. Breads were whole grain breads that contained seeds to increase the protein content. Snacks were mostly dairy products. PG was offered altogether approximately 27.5 g/d more protein than NG, of which 4.3 g was provided by the main meals, 10.6 g by snacks and 7.2 g by bread. Protein supplements were not used. The Control group (CG) continued with their normal diet without any meal service. The nutritional composition of the intervention meals has been previously reported [13].

### 2.3 Measurements

Nutritional status was assessed by the Mini Nutritional Assessment (MNA). Nutrient intake was assessed by a three-day food diary which was analyzed using the Finnish National Food Composition Database, Fineli [14]. Three-day food diaries were collected at baseline and at the end of the study. The amounts and the quality of food consumed were confirmed by check calls.

The three-day food diaries were used to assess the mean daily protein intake and the mean cost for daily diet. The cost for daily diet, including offered meals and other foods and drinks, that participants consumed, were assessed from the food diaries collected at the end of the study. Cost for food items was calculated using the database from foodie.fi, a database of the S Group, an organization whose market share of Finland’s grocery store selling was the highest (46,4 %) in 2018 when the food diaries were collected [15]. If the name of the product was mentioned in the food diary, the actual price was used. If it was not, the cheapest price for that item was used. Prices of alcoholic beverages were collected from Alko’s online store (national alcoholic beverage retailer). Cost for the meals provided by the food service included only the price of the meal without any delivery costs. Prices included value added tax (VAT). Daily cost was calculated as the mean cost of the three food diary days. The cost was assessed by the perspective of the participants, as they are home-dwelling older adults, who would use and buy these kinds of food and products home.

Incremental cost effectiveness ratio (ICER) was used to assess cost effectiveness. An ICER is a summary measure representing the economic value of an intervention, compared with an alternative. It is used to summarize the results of economic evaluations of health interventions [16]. It is calculated by dividing the difference in total costs by the difference in the chosen measure of a health outcome or effect to provide a ratio of extra cost per extra unit of health effect, for the more expensive therapy vs. the alternative. It indicates which option provides the highest value for the cost.

### 2.4 Statistical analyses

Differences between the groups were evaluated using the one-way analysis of variance (ANOVA) for normally distributed continuous variables and the Kruskal–Wallis test for non-normally distributed variables. Changes within the group were analyzed using paired samples T-test for normally distributed variables and Wilcoxon Signed Rank Test for non-normally distributed variables and for categorical variables.

Cost analyses were performed using a generalized linear regression model with log link and gamma variance functions. The variance function was selected based on Park test and Akaike's information criterion. The relationship between protein intake and daily cost ratios was estimated using bias-corrected bootstrapping (5 000 replications).

## 3 Results

### 3.1 Nutritional status and nutrient intake

At the end of the Power Meals study, 34.4. % of the participants were at risk of malnutrition (MNA score 17–23.5). The change in MNA score was significant in the PG, from 24.5 to 26.7 (p = 0.024), but not in NG or CG (Table 1).

**Table 1:** Nutritional status, protein intake and energy intake at baseline and at the end of the intervention.

	PG (n = 22)			NG (n = 23)			CG (n = 20)		
	Baseline	8 week	P-value*	Baseline	8 week	P-value*	Baseline	8 week	P-value*
**Nutritional status**									
MNA points, mean ± SD	24.5 ± 3.3	26.7 ± 2.20	**0.024**	24.5 ± 3.2	25.1 ± 2.2	0.504	23.8 ± 2.9	23.5 ± 2.8	0.597
**MNA categories, n (%)**									
Normal nutritional status, >23.5 p.	16 (72.7)	17 (77.3)	0.317	16 (69.6)	16 (72.7)	1.000	12 (60.0)	9 (45.0)	0.414
Risk of malnutrition, 17−23.5 p.	5 (22.7)	5 (22.7)		7 (30.4)	6 (27.3)**		7 (35.0)	11 (55.0)	
Malnourished, <17 p.	1 (4.5)	–		–			1 (5.0)	–	
**Protein intake**									
g/ikg/d, mean ± SD	0.92 ± 0.38	1.03 ± 0.27	0.064	0.94 ± 0.31	0.91 ± 0.22	0.620	0.93 ± 0.28	0.88 ± 0.20	0.352
g/d, mean ± SD	65.4 ± 23.5	74.8 ± 14.9	**0.032**	69.4 ± 25.6	66.0 ± 13.1	0.481	68.0 ± 21.4	64.4 ± 16.8	0.347
**Energy intake**									
kcal/d, mean ± SD	1582 ± 564	1767 ± 343	**0.044**	1589 ± 486	1736 ± 409	0.150	1579 ± 425	1532 ± 368	0.603

PG = Protein group, NG = Normal group, CG = Control group, SD = Standard Deviation. MNA = Mini Nutritional Assessment. g/ikg/d = grams per ideal body weight per day. If the participant’s body mass index (BMI) was between 20 and 30 kg/m^2^, the actual BMI was used. If BMI was under 20 kg/m^2^, it was adjusted to 20 and if it was above 30 kg/m^2^, it was adjusted to 30.

* Change within the group was tested with Paired samples T-test for normally distributed variables and Wilcoxon Signed Rank Test for non-normally distributed variables and for categorical variables.

** n = 22.

Protein intake (g/d) increased in PG (p = 0.032). Changes in NG and CG were not statistically significant (Table 1). The change for energy intake was significant in PG where energy intake increased from 1582 ± 564 kcal to 1767 ± 343 kcal (p = 0.044). Changes in NG and in CG were not statistically significant.

### 3.2 Cost for daily diet

Mean cost for the daily diet was 8.35 ± 1.61 € (min.4.38 €, max. 10.58 €) in PG, 7.94 ± 1.68 € (4.70–11.85 €) in NG and 5.65 ± 1.77 € (2.84–9.63 €) in CG (Table 2). Cost of the daily diet in PG and NG did not differ significantly from each other, but both differed significantly from CG (p < 0.001).

**Table 2:** Protein intake, daily cost, incremental cost-effectiveness ratios (ICER) and effect of increasing the cost in the study groups.

Group	Protein intake, g/ikg/d, mean ± SD	Cost per day €/d mean ± SD	Cost-effectiveness ratios*, mean (95% CI)	Effect on protein intake (g/ikg/d) when increasing the cost by one euro** (95% CI)
PG	1.03 ± 0.27	8.35 ± 1.61	8.11 (7.28–8.94)	B = 0.048 (-0.005 to 0.101) p = 0.076
NG	0.91 ± 0.21	7.94 ± 1.68	8.72 (7.84–9.76)	B = 0.005 (-0.043 to 0.053) p = 0.830
CG	0.88 ± 0.19	5.65 ± 1.77	6.45 (5.82–7.27)	B = 0.062 (0.016 to 0.109) p = 0.009

PG = Protein group, NG = Normal group, CG = Control group, CI = Confidence interval.

* The cost of daily diet in proportion to daily protein intake. The ratio was estimated using bias-corrected bootstrapping (5 000 replications).

** Adjusted for age and sex.

### 3.3 Cost-effectiveness for increasing protein intake

Incremental cost-effectiveness analysis showed that increasing protein intake was cost-effective in PG as the cost-effectiveness ratio in PG was lower than in NG (Table 2). Increasing the daily cost by one euro could significantly increase the protein intake in CG (p = 0.009) but not in PG (p = 0.076) or in NG (p = 0.830). However, the cost in CG was significantly lower than in other groups (p < 0.001) so the change is presumable.

## 4 Discussion

This study shows that a protein rich diet can be more expensive than the normal diet that older adults usually follow. Increasing protein intake by protein rich foods and snacks is more cost-effective than efforts to increase protein intake by normal home meals, although protein rich food is often more expensive.

The cost for the daily diet in the present study varied from 5.65 euros in CG to 8.35 euros in PG. Finnish Faculty of Social Sciences has composed a referential budget for a reasonable minimum of dietary costs in 2021 and according to it, Finnish women and men aged over 65 years spend 185 euros and 212 euros, respectively, for food per month [17]. This would mean approximately 5.5 and 6.4 euros per day, which is in line with the cost in CG. A Swedish study found in 2008 that the average cost of a daily diet was 6.95 euros [18] and a Dutch study found it to be 5.31 euros in 2019 [19].

There are only few cost-related European studies [20]. An American study found in 2010 that protein content of the diet was associated with higher food costs [21]. Multiple studies have found high nutrient quality diets to be associated with higher diet costs [[22], [23], [24], [25]]. These findings are in line with the present study as the cost was the highest in PG. The cost was assessed with food diaries and supermarket prices. Merging supermarket food prices with standard dietary assessment tools, such as food diaries, has found to be a good way to provide estimates of individual diet costs that are closely associated with the food consumed [26].

Increasing protein intake by protein rich foods and snacks was found to be cost-effective in the present study. Food-based nutrition interventions and personalized dietary advice have been found to be cost-effective [8,9], but the present study used only meals and snacks instead of dietary advice. Snacks were important as they provided 16 g of the 27.5 g extra protein that PG consumed. Snacks were mostly dairy products such as quark and milkshakes. These products were not typical in the participants’ normal diet. Introducing these kinds of new products could be useful when trying to increase protein intake in older adults and dairy-based snacks can also help to improve nutritional status and quality of life [27,28]. Individually tailored nutritional guidance can improve protein intake and it is also found to be cost-effective [10,29].

The strength of this study is that it provides important new evidence of cost-effectiveness, since there are only few cost-effectiveness analyses concerning protein intake among older people. Another strength is, that the Power Meals study was a randomized, controlled study, that used only regular food items instead of artificial products as older people might be suspicious with artificial products. This may increase the reliability of the cost-effectiveness analysis, as the effectiveness has been assessed with commonly accepted foods. This study has some limitations. The sample size was small with 65 participants. We did not use the cost-effectiveness acceptability curve (CEAC) to explore the uncertainty of the results. As always with food diaries, there could have been some inaccuracies with reporting, although they were checked by phone. We used prices from only one organization, although it is the biggest one in Finland. We must also notice that the food prices have risen since this analysis, but it is unlikely that this affects the difference in the costs between groups. In the future it could be beneficial to study cost-effectiveness including delivery costs of home-delivered meals.

## 5 Conclusion

Including protein-rich meals and snacks in a diet is a cost-effective way to increase protein intake in home-dwelling older adults. Introducing protein-rich products as a part of dietary advice and meal services could be an effective way to increase protein intake.

## Conflict of interests

Mrs. Rautakallio-Järvinen reports payments from Oy Karl Fazer Ab during the price analyses but not during the preparation of this article. Dr. Laaksonen and Mrs. Fogelholm report payments from Oy Karl Fazer Ab or Compass Group FS Finland as employees during the preparation of this article.

## Ethical standards

The study was approved by the Ethics Committee of the Hospital District of Southwest Finland.

## Declaration of generative AI and AI-assisted technologies in the writing process

AI or AI-assisted technologies were not used in the writing process of this article.
